# Relations between family cohesion and adolescent-parent’s neural synchrony in response to emotional stimulations

**DOI:** 10.1186/s12993-022-00197-1

**Published:** 2022-09-27

**Authors:** Xinmei Deng, Mingping Lin, Lin Zhang, Xiaoqing Li, Qiufeng Gao

**Affiliations:** 1 School of Psychology, Shenzhen University, Shenzhen, China; 2 Department of Society, School of Government, Shenzhen University, Shenzhen, China

**Keywords:** EEG hyperscanning, Family cohesion, Adolescents, Interbrain synchrony, Emotional processing

## Abstract

**Background:**

The interaction between parent and adolescent is more challenging than in other age periods. Family cohesion seriously impacts parent-adolescent emotional interactions. However, the underlying neural mechanism has not been fully examined. This study examined the differences in the neural synchrony in response to emotional film clips between high and low family cohesion adolescent-parent dyads by using the electroencephalograph (EEG) hyperscanning.

**Results:**

Simultaneously electroencephalograph (EEG) was recorded while 15 low family cohesion parent-adolescent dyads (LFCs)and 14 high family cohesion parent-adolescent dyads (HFCs)received different emotional induction when viewing film clips. Interbrain phase-locking-value (PLV) in gamma band was used to calculate parent-adolescent dyads’ interbrain synchrony. Results showed that higher gamma interbrain synchrony was observed in the HFCs than the LFCs in the positive conditions. However, there was no significant difference between the HFCs and LFCs in other conditions. Also, the HFCs had significantly higher gamma interbrain synchrony in the positive conditions than in the negative conditions.

**Conclusion:**

Interbrain synchrony may represent an underlying neural mechanism of the parent-adolescent emotional bonding, which is the core of family cohesion.

## Introduction

Family cohesion refers to the emotion, support, helpfulness and caring among family members [41]. Adolescence can be considered a sensitive period for social development, with psychological and social cognitive changes [6]. During adolescence, the social world and interpersonal interactions become increasingly important. Previous research indicated that family cohesion may help adolescents to overcome the challenges of social interactions by receiving social and emotional support from family members [24]. However, the underlying neural mechanism for the impacts of family cohesion on adolescent-parent emotional interactions has not been fully examined. As a biomarker of social and emotional interaction, the electroencephalogram (EEG) interbrain synchrony may represent the behavioral and emotional synchrony, which reflects the emotional bond within adolescent-parent dyads [28]. Making further exploration of the impacts of family cohesion on the neural synchrony within adolescent-parent dyads during an emotional related task may shed light on the understanding of the biological base of social interaction. Therefore, this study examined the differences in the neural synchrony in response to different emotional stimuli simultaneously between the adolescent-parent dyads who had different level of family cohesion.

Family cohesion is defined as the emotional bonding that family members have toward each other [42]. It is the core component of family functioning. Previous studies indicated that balanced level of family cohesion (e.g., from moderately low to moderately high level) is beneficial and viable for healthy family functioning [41]. In a functional and cohesive family, family members can be emotionally connected to, provide support and care for their families [49].

Family cohesion has significant impacts on adolescents’ behavior, socio-emotional development, well-being in their later life, and caregivers’ parenting behaviors [5, 43]. It is highly related to individuals’ positive well-being. For example, adolescents in families with a high level of family cohesion would report more positive emotional experiences, more life satisfaction, and more meaning and purpose in life [17]. Adverse family processes may operate to increase adolescents' vulnerability to depression [48]. Previous research also indicated that family cohesion is associated with a lower level of negative behavioral outcomes during development (e.g., externalizing behaviors) [20]. The influence of family cohesion on children and adolescents’ behavioral problems may be moderated by the caregivers’ health conditions and parenting behaviors. Family cohesion can provide important emotional support and boding to reduce the negative impacts of maladaptive parenting behavior on children’s behavioral problems [31]. It is considered to be an important protective buffer as it meets the individual’s psychological need for affiliation, security and emotional connection with others [46].

Not only the positive impacts on adolescents’ behavior and mental health, family cohesion also has protective impacts on social and emotional interaction for adolescents. Family cohesion is positively correlated with close emotional bonding and depend attachments. A cohesive family reflects a symbiotic and interdependent relationship between family members [1]. For example, a child’s mental and emotional status would depend on and be impacted by the other members. Family cohesion is also related to the family member’s feelings (i.e., loneliness), which reflect their family environment and the quality of the interactions with other family members [24]. Previous research indicated that family cohesion is negatively correlated with the parent-adolescent conflict [52]. Higher family cohesion which is characterized as a higher family engagement is negatively correlated with the adolescents’ depressive symptoms, higher levels of family support, and less psychological control from mothers [49]. Adolescents who grow up in a family with moderate cohesion levels would have more positive communication and are more comfortable to be close with other during social interactions. Adolescents with high family cohesion would show better communication skills and feel less anxious and negative experiences during socio-emotional interactions [40]. Previous research suggested that family cohesion is positively associated with parental support, responsiveness, and greater positive parent-child engagement. Family members have significantly more contact and communication with one another. It is possible that the family rules in a cohesive family make family members stay more in contact with one another [44]. On the contrary, a lack of family cohesion is associated with negative parent-child interaction which may increase social stress and reduce emotional support from the parents during social interaction [21]. Family members with low cohesion would have less involvement in one another’s lives and show less emotional bonding with one another [40].

Adolescence is characterized as increased independence and the development of self. With the pursuit of independence out of the family environment, adolescents spend more time alone or with their peers, and the conflict affective intensity interactions between adolescents and their parents increase [50]. As stated above, family cohesion may serve as a protective factor for parent-adolescent conflicts during socio-emotional interactions and seriously influence the effectiveness and quality of parent-adolescent emotional interactions [24]. The most important impact of family cohesion could increase the psychological and emotional bonding between parent-adolescent dyads. During socio-emotional interactions, the behavioral, emotional, physiological and neural synchronization has been considered as an evolutionary adaptive outcome of interpersonal bonding [22]. In this case, when engaging in social situations, family members in a high cohesive family might present a higher level of similarities in behavioral, emotional, physiological and neural levels because of their inherently biological and emotional bonding. However, there is little neural evidence to suggest that the level of family cohesion is associated with neural interbrain synchrony between adolescents and parents when experiencing positive and negative emotions together.

As the most important social relationship of each individual, the interaction between infant and caregiver is thought to be the first experience of social interaction. The interbrain synchrony between infant and caregiver in biological rhythms and social signals reflects the inherently biological and emotional bonding, which is also an important feature of the early brain matures [15]. Previous research demonstrated that the moment-to-moment interbrain synchrony is a sensitive maker that can predict dynamic socio-emotional interactions [14]. It is likely driven by the shared attention and emotional processing mechanism within the dyads during moments of social contact and communication.

Generally, there are four types of interbrain synchrony, induced synchrony is associated with the phenomenon that two brains tend to be synchronized with the influence of common external stimuli [8]. For instance, two participants will show synchrony without information transmission and interaction, such as viewing a movie [19]. According to the phase resetting theory, each individual is sending out conspicuously social signals in the process of social interaction, such as gaze, voice, body posture. As the trigger of neural synchrony, these signals trigger the phase reset of the ongoing neural oscillations of the interacting parties, thus causing the phase difference between the two signal strings to remain constant [53]. Previous research suggested that interbrain synchrony, which can be measured by the EEG-based functional connectivity provides a useful tool for studying the simultaneous brain activity between dyads during interactions in different emotional states [11]. For example, in a study, participants viewed film clips that evoked different emotional states (e.g., neutral, positive, or negative). There was an overall increase of interbrain synchrony during emotional stimulation (e.g., during viewing positive or negative film clips). However, the interbrain synchrony indices were significantly different among emotional states [30]. Another study also found that there was a significant difference of hyper-connectivity existed in the gamma frequency band between positive and negative stimulus conditions [55]. Interbrain synchrony between mother and child was found in the medial left cluster of the prefrontal cortex in a dyadic task of watching animation [2]. During social interactions, compared with the negative emotional states, the inter-brain network showed significantly higher strength for positive emotional states for the parent-infant dyads [47].

This biobehavioral synchrony could be a consequence of the coordination of physiological and behavioral processes when individual and their caregiver engage in each other’s social life [15]. Increased interbrain synchrony facilitates emotional sharing, social understanding, psychological support, empathy between individual and their caregiver because of the accessibility to each other’s internal state [54]. In this case, such interbrain synchrony could be an important neural indicator of the cohesion level for the family members.

The quality and connection between parents and adolescents during social interaction and social emotion perception could reflect the level of family cohesion and family atmosphere. However, little is known about the neural underpinnings about how the level of family cohesion is associated with the interpersonal neural connectivity between adolescents and parents. As mentioned above, induced synchrony could be reflected from the phenomenon that two brains tend to be synchronized with the influence of common external stimuli [8]. Shared emotional experiences can induce interbrain synchrony between parent and child because of the formation of parallel attuned emotional responses [2]. Thus, in the current study, the hyperscanning method was used to examine the neural synchrony in response to different emotional film clips between adolescent-parent dyads in different level of family cohesion. Because of the positive impacts of family cohesion on socio-emotional interactions between family members [27], we hypothesized that high family cohesion parent-adolescent dyads would have high levels of interbrain synchrony in response to emotional film clips. Moreover, previous study demonstrated that the hyper-connectivity (e.g., in the gamma frequency band) in the positive stimulation condition was greater than in the negative stimulation condition [55]. We hypothesized that how the level of family cohesion associated with the interbrain synchrony between adolescents and parents would be different in the positive and negative emotional conditions.

## 2 Methods

### 2.1 Participants

Adolescent participants were recruited via flyers that invited healthy volunteers to participate in a study of parenting and emotion. Interested families were invited to visit the university laboratory to take part in the study. Final samples of the study were recruited on the basis of their scores on the Family Adaptability and Cohesion Scale, Chinese Version, (FACESII-CV; [16]). The 16-item Family Cohesion Subscale of FACESII-CV was used to assess parent-adolescent dyads’ level of family cohesion and emotional bonding among family members on a 5-point Likert scale (1= never or very rarely true, 5= very often or always true; e.g., “All the family members get together for activities”). (α = .71).

Given the poor EEG data quality and technical error, 2 parent-adolescent dyads were excluded from the study. Thus, the final sample was composed of 15 low family cohesion parent-adolescent dyads (LFCs; 11 male and 4 female, aged from 11 to 14 years old, Mage=12.00, *SD*=1.25, 6 mothers, *M*_age_=43.56, *SD*=5.43; 9 fathers, *M*_age_=44.22, *SD*=4.63) and 14 high family cohesion parent-adolescent dyads (HFCs; 11 male and 3 female, aged from 11 to 14 years old, *M*_age_=12.36, *SD*=1.08, 7 mothers, *M*_age_=42.57, *SD*=1.90; 7 fathers, *M*_age_=42.76, *SD*=3.21). HFCs (*M*_HFCs_=76.93, SD=3.71) and LFCs (*M*_LFCs_=66.33, *SD*=3.64) differed significantly on the overall family cohesion score (*p*<.001). Optimal sample size calculations using G*Power (Faul, et al., 2007) for a medium effect size repeated measures design (groups = 2, *f* = .25, a = .05, 1- beta = .80) resulted in total *N* = 24. The sample size in this study was also in line with typical hyperscanning EEG studies [9, 10, 32].

All of the adolescents came from urban communities in Shenzhen city in China. Approximately 93.10% of fathers and 89.66% of mothers had received a college education, whereas other parents had received an education of high school or lower. All of the participants were right-handed and had normal or corrected-to-normal vision, and were in good neurological and psychiatric condition. No participant had a history of neurological or psychiatric disorder, as determined by self- and/or parent report. The research protocol was approved by the local Institutional Review Board. All procedures performed in studies involving human participants were in accordance with the ethical standards of the institutional and/or national research committee and with the 1964 Helsinki declaration and its later amendments or comparable ethical standards. Informed consent was obtained from the participants and their parents before the study, and the parent-adolescent dyads were fully debriefed after the experiment.

### 2.2 Stimuli

Six film clips were selected from the Chinese Affective Film System(CAFS; Xu et al, 2010). The film clips were age-appropriate for adolescents. Negative film clips consisted of 2 100-s segments from “My Beloved” and “My Sisters and Brothers”. The negative film clips were previously found to elicit a strong level of sadness (between 4 and 5 on the 5-point rating scale, *M*=4.5) with low variation across subjects (*SD* between 2 and 3, mean SD 2.5). The negative film clips included unpleasing social situations, such as being separated from loved ones. For example, in the film clip of “My Sisters and Brothers”, the older brother had to give his youngest sister to others for adoption because of poverty. The youngest sister cried out and begged her brother not to abandon her. Positive film clips consisted of 2 100-s segments from “A big potato” and “Eat Hot Tofu Slowly”. The positive film clips were previously found to elicit a strong level of happiness (between 4 and 5 on the 5-point rating scale, *M*=4.5) with low variation across subjects (*SD* between 1 and 2, mean *SD* 1.5). For example, in the film clip of “Eat Hot Tofu Slowly”, a student sleeping in class was asked to sing a song by the teacher. Because his singing was out of tune, the whole class laughed. Neutral film clips consisted of 2 100-s segments from “Computer Maintenance” and “IP Encapsulation and IDE Interface Fixes”. The neutral film clips were previously found to elicit a medium level of emotional intensity (between 1 and 4 on the 5-point rating scale, *M*=1.5) with low variation across subjects (*SD* between 1 and 2, mean *SD* 1.5). The neutral film clips included one person completes the operation of repairing the computer and repairing the IDF interface. For example, in the film clip of “IP Encapsulation and IDE Interface Fixes”, an engineer turns the screws, removes the keyboard and trackpad, checks the wires and so on.

### 2.3 Procedures

We employed a 2 (Group: HFCs vs. LFCs) ×3 (Valence: positive vs. negative vs. neutral) repeated measures design. After receiving the demographic information from the parent-adolescent dyads, the parent-adolescent dyads were asked to see the film clips together. Electroencephalograph (EEG) sensors were attached on both of the parent-adolescent dyads and they were introduced to the procedures of the task before the experiment. After the research assistant confirmed the parent-adolescent dyads fully understood the procedure, the experiment began. At the beginning of each trial, a fixation point was presented for one minute in the middle of the screen. Following the fixation, a film clip was presented for 100s. Then, participants were asked to rate each film clip on the valence and arousal scales of the self-assessment manikin (SAM, [29] on the computer by keyboards, ranging from low pleasure or arousal (1) to high pleasure or arousal (5). The parent-adolescent dyads were told to rate the valence of each film clip based on how pleasant the film clip made them feel respectively (e.g., How pleasant did you feel after viewing the film clip?). Then, the parent-adolescent dyads were told to rate arousal based on the strength of their feelings in response to the film clip respectively (e.g., How strongly did you feel after viewing the film clip?). The valence and arousal ratings were not shown on the screen. Parents and adolescents can’t see each other’s ratings.

**Fig. 1:**
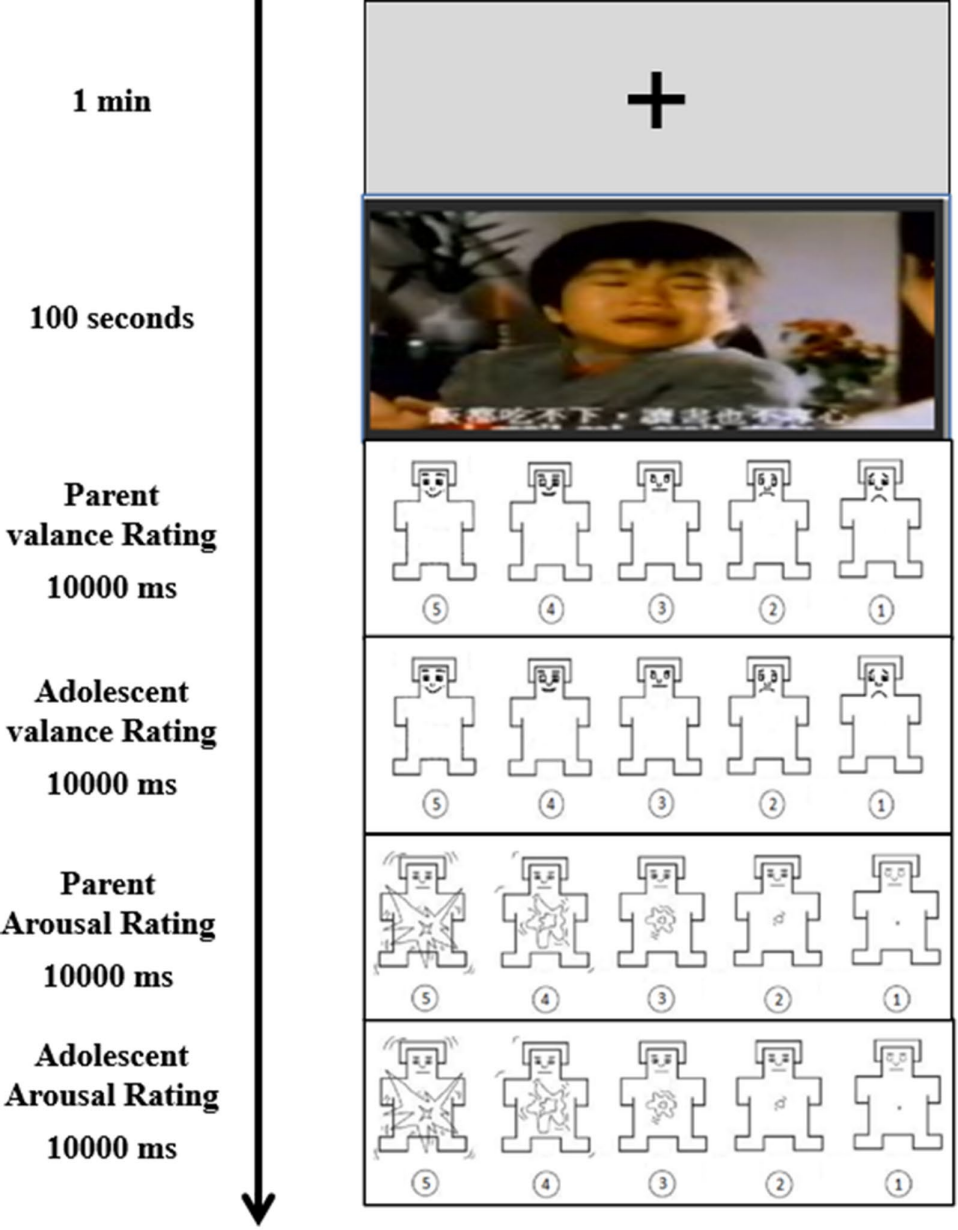
Sample of stimulus and procedure

There were six film clips in the experiment. Each film clip was displayed once in random order. E-Prime software was used to present all stimuli against a black background on a 21-inch monitor, with a viewing distance of approximately 80 cm. An experimental session took 30-35 min for each participant (see Figure 1). The parent-adolescent dyads were fully debriefed the study after the whole study.

### 2.4 Dual-EEG recording and data analysis

The parent-adolescent dyads were comfortably seated side by side in an electrically shielded and dimly lit room. Simultaneously and continuously EEG signals of each dyad during viewing six film clips were recorded using two 32-channel portable EEG systems (BrainAmp, Brain products GmbH, Germany) at a sampling rate of 500 Hz. Electrode impedance was kept under 10 kohms for all recordings. Online reference electrodes were placed at TP9 on the parents’ head. EEG signals were referenced offline to the averaged mastoid references and band-pass filtered in the range of 1 to 40 Hz. Epochs with eye movement or other movement artifacts were manually removed by inspection. Independent component analysis (ICA) was performed for ocular artifact reduction was performed by using EEGLAB. The artifact scored epochs were eliminated from all subsequent analyses. Onsets were set as the points where the film clips started. The artifact-free EEG signal from each trial was segmented from 0s before to 100 s after the onset of the film clips. The film clips were segmented into 1-s epochs in order to provide sufficient samples for a more faithful classification accuracy estimation [23]. The mean number of valid epochs for LFCs was 173.40 (*SD*=24.7) for positive stimuli, 184.07 (*SD*=26.13) for negative stimuli and 174.27 (*SD*=29.03) for neutral stimuli. The mean number of valid epochs for HFCs was 172.93 (*SD*=34.52) for positive stimuli, 180.29 (*SD*=34.78) for negative stimuli and 177.57 (*SD*=25.72) for neutral stimuli

In previous studies, inter-brain synchrony has been measured by the phase synchronization of EEG signals [47]. We used an interbrain phase-locking-value (PLV) index to estimate the interbrain phase synchrony between parent-adolescent dyads in the positive, negative and neutral conditions. The interbrain phase synchrony index has been developed to measure whether the signals from the two interacting individuals are phase locked across time [12]. In line with previous hyperscanning research [4], band-pass filtering is applied to the EEG data of each segment of the parent’s electrode (φ) and the adolescent’s electrode (ψ) to obtain the EEG data of the frequency band of interest. The Hilbert transform is used to analyze the signals of φ and ψ poles in a specific frequency band, extract phase and subtract, and get the PLV of each epoch. Average the PLV of electrodes φ and ψ in the frequency band of interest is obtained by average for epoch under each condition. Therefore, PLV can be expressed by the formula, where N is the number of trials, φ_(t, n)_ is the phase on trial, n at time t, in channel φ and ψ_(t, n)_ in channel ψ. The averaged interbrain PLVs in gamma frequency bands (31-40Hz) in the frontal (Fz), central (Cz) and parietal areas (Pz) were used for the inter-brain synchrony analyses. The averaged interbrain PLVs in the following four frequency bands were calculated for further statistical analysis: delta (1-3 Hz), theta (4-7 Hz), alpha (8-13 Hz), beta (14-30 Hz). Results of the averaged interbrain PLV in other frequency bands were shown in Appendix A. The frequency band has been considered to be highly related to emotional interaction [26, 39]. PLVs were averaged across the positive, negative and neutral trials.

$${\mathrm{PLV}}_{\mathrm{t}}={\mathrm{T}}^{-1}|\sum_{\mathrm{n}=1}^{\mathrm{T}}{\mathrm{e}}^{\mathrm{i}\left[\mathrm{\varphi }\left(\mathrm{t},\mathrm{n}\right)-\Psi (\mathrm{t},\mathrm{n})\right]}|$$

The interbrain PLV was assessed using a 2 (group: HFCs vs. LFCs) × 3 (valence: positive vs. negative vs. neutral) × 3 (electrode: Cz vs. Fz vs. Pz) repeated measures ANOVAs, with valence and electrode as within-subject variable and group as between-subject variable. The interbrain PLV was statistically evaluated using SPSS 20.0. Greenhouse-Geisser’s method was adopted to correct for violations of sphericity. For the post-hoc comparisons, two-sample t-tests were used. Two-tailed test with a p-value of less than .05 was used to determine the level of significance. Bonferroni method was used to correct for multiple comparisons in the post-hoc analyses, respectively. Partial eta squared was reported as a measure of effect size.

To rule out the possible impact of relevant variables of the parent-adolescents emotional interactions on our findings, demographic variables, adolescents’ level of depression, adolescents’ level of anxiety, adolescents’ level of social support, parents’ level of depression, parents’ level of anxiety, and the level of parent involvement between the high and low social anxiety parent-adolescents dyads were examined. Repeated measures ANOVAs were used and these relevant variables were set as the covariates. The results of the repeated measures ANOVA indicated that there were no significant main effects of demographic variables and other examined variables on the interbrain PLV between the high and low social anxiety groups (see Appendix B).

## Results

### Behavioral results

Table 1 showed the average valence ratings and average arousal ratings of the parent-adolescent dyads when viewing positive, negative and neutral emotional films.

**Table 1:** Average Valence Ratings and Arousal Ratings between Low and High Family Cohesion Parent-adolescent Dyads in Different Conditions

	Valance	Dyad	LFCs (*M* ± *SD*)	HFCs (*M* ± *SD*)	*t(27)*	*d*	*p*
Average valence ratings	Positive	Parent	3.90 ± .71	4.22 ± .42	− 1.44	− 0.55	0.162
		Child	3.78 ± .96	3.93 ± .69	− 0.48	− 0.18	0.634
	Negative	Parent	2.18 ± .96	1.83 ± .73	1.10	.41	0.283
		Child	2.83 ± .76	2.89 ± 1.05	− 0.17	− 0.07	0.863
	Neutral	Parent	2.49 ± .82	2.52 ± .60	− 0.12	-.04	0.906
		Child	2.51 ± .78	2.59 ± .55	− 0.31	− 0.11	0.756
Average valence ratings	Positive	Parent	3.94 ± .50	4.22 ± .87	− 1.07	− 0.39	0.292
		Child	3.36 ± .48	3.77 ± .94	− 1.51	− 0.55	0.141
	Negative	Parent	2.92 ± 1.03	2.99 ± .71	− 0.21	− 0.08	0.837
		Child	3.15 ± .87	3.47 ± 1.00	− 0.91	− 0.34	0.371
	Neutral	Parent	2.23 ± .90	2.75 ± .96	− 1.50	− 0.56	0.146
		Child	1.96 ± .80	2.32 ± .56	− 1.40	− 0.52	0.173

*LFCs* low family cohesion parent-adolescent dyads, *HFCs* high family cohesion parent-adolescent dyads

We employed a 2 (Group: HFCs vs. LFCs) × 3 (Valence: positive vs. negative vs. neutral) × 2 (Dyad: parent vs. adolescent) repeated measures ANOVA to analyze the differences in the average valence rating. The main effect of Valence was significant, *F*(2, 108) = 63.27, *p*< .001, *η*_*p*_^*2*^= .54. The valence ratings of the positive film were higher than the negative and neutral films (both *p*< .001). The main effect of Dyad was significant, *F*(1, 54) = 4.86, *p* = .032, *η*_*p*_^*2*^ = .83. The valence ratings of parents were significantly lower than the valence ratings of adolescents (*p* = .032). The main effect of Group was not significant, *F*(1, 54) = .21, *p* = .645, *η*_*p*_^*2*^ = .00.The interaction of Dyad and Valence was significant, *F* (2,108) =6.65, *p*=.005, *η*_*p*_^*2*^=.11.The valence ratings of parents were significantly lower than the valence ratings of adolescents in the negative conditions. (*p* = .001). However, there was no significant difference between parents and adolescents in the positive (*p* = .280) and neutral conditions (*p* = .810). The interaction of Group and Valence was not significant, *F* (2,108) =.77, *p*=.433, *η*_*p*_^*2*^=.010. The interaction of Group and Dyad was not significant, *F* (1,54)=.21, *p*=.646, *η*_*p*_^*2*^=.00. The interaction of Group, Dyad, and Valence was not significant, *F* (2,108)=.45, *p*=.585, *η*_*p*_^*2*^=.01.

We employed a 2 (Group: HFCs vs. LFCs) × 3 (Valence: positive vs. negative vs. neutral) × 2 (Dyad: parent vs. adolescent) repeated measures ANOVA to analyze the differences in the average arousal rating. The main effect of Valence was significant, *F* (2, 108) = 63.10, *p*< .001, *η*_p_^*2*^= .54. The arousal ratings of the positive films were higher than the negative and neutral films (both *p*<.001). The arousal ratings of the negative films were higher than neutral films (*p*<.001). The main effect of Dyad was not significant, *F*(1, 54) = 1.26, *p* = .267, *η*_*p*_^*2*^ = .02. The main effect of Group was significant, *F*(1, 54) = 4.74, *p* = .034, *η*_*p*_^*2*^ = .81.The arousal ratings of LFCs were significantly lower than the arousal ratings of HFCs (*p* = .034). The interaction of Dyad and Valence was significant, *F* (2,108)=5.89,*p*=.004, *η*_*p*_^*2*^=.10. The arousal ratings of adolescents were significantly lower than the arousal ratings of parents in the positive conditions. (*p* = .009). However, there was no significant difference between parents and adolescents in the negative (*p* = .144) and neutral conditions (*p* = .113). The interaction of Group and Valence was not significant, *F* (2,108)=.43, *p*=.651, *η*_*p*_^*2*^=.01.The interaction of Group and Dyad was not significant, *F* (1,54)=.07, *p*=.800, *η*_*p*_^*2*^=.00.The interaction of Group, Dyad, and Valence was not significant, *F* (2,108)=.30, *p*=.745, *η*_*p*_^*2*^=.01.

To examine the similarity in valence and arousal ratings between the parents and adolescents, Pearson’s correlation coefficients were calculated between the valence ratings and arousal ratings of the parents and adolescents. Fisher’s z transformation was conducted for the Pearson’s correlation coefficients before the t-test for rating similarities. Independent t-tests were conducted to examine the differences in the similarity in valence and arousal ratings between high and low family cohesion dyads. As show in Table 2, there was no significant difference in the similarity in valence (*p* = .964) and arousal ratings (*p* = .748) between high and low family cohesion dyads.

**Table 2:** Similarity in the Valence and Arousal Ratings between High and Low Family Cohesion Dyad*s*

	Group	(*M* ± *SD*)	*t(27)*	*d*	*p*	95%CI	
Valence ratings	LFCs	0.40 ± 0.40	− 0.05	0.02	0.964	− 0.79	0.76
	HFCs	0.41 ± 0.51					
Arousal ratings	LFCs	0.35 ± 0.41	− 0.33	-0.13	0.748	− 0.90	0.65
	HFCs	0.39 ± 0.35					

95%CI = 95%CI for the similarity difference in the valence and arousal ratings between high and low family cohesion parent-adolescent dyads

### Neural results

To examine the interbrain synchrony between the parent-adolescent dyads when viewing positive, negative and neutral emotional films, we calculated the interbrain phase-locking-value (PLV) (as shown in Table 3) which has been developed to measure whether the signals from the two interacting individuals are perfectly phase-locked across time.

**Table 3:** Gamma Interbrain Phase-locking-value (PLV) between Low and High Family Cohesion Parent-adolescent Dyads in Different *Conditions at Fz, Cz and Pz*

Electrode	Conditions	LFCs (*M* ± *SD*)	HFCs (*M* ± *SD*)	*t (27)*	*d*	*p*	95% CI for the PLV differences between LFCs and HFCs	
Fz	Positive	0.225 ± 0.006	0.221 ± 0.011	1.30	0.45	0.205	− 0.003	0.011
	Negative	0.222 ± 0.014	0.224 ± 0.010	− 0.42	− 0.16	0.676	− 0.011	0.007
	Neutral	0.225 ± 0.006	.221 ± 0.011	− 0.90	0.45	0.373	− 0.010	0.004
Cz	Positive	0.225 ± 0.009	.221 ± 0.011	0.99	0.40	0.333	− 0.004	0.011
	Negative	0.219 ± 0.008	.220 ± .011	− 0.48	-0.10	0.635	− 0.010	0.006
	Neutral	0.223 ± 0.012	.219 ±0 .011	0.62	0.35	0.544	− 0.006	0.012
Pz	Positive	0.220 ± 0.005	0.229 ± 0.008	− 3.52	− 1.35	0.002	− 0.015	− 0.004
	Negative	0.223 ± 0.011	0.218 ± 0.011	1.10	0.45	0.282	− 0.004	0.013
	Neutral	0.222 ± 0.014	0.225 ± 0.013	− 0.54	− 22	0.593	− 0.013	0.008

*LFCs* low family cohesion parent-adolescent dyads, *HFCs* high family cohesion parent-adolescent dyads

Results of the repeated measures ANOVA on the interbrain phase synchrony in the gamma band showed that the main effect of Group was not significant, *F*(1,27)= .04, *p*=.852, *η*_*p*_^*2*^= .001. The main effect of Valence was not significant, *F* (2, 54)=1.43, *p*=.249, *η*_*p*_^*2*^= .05. The main effect of Electrode was also not significant, *F*(2, 54)=.79, *p*=.430, *η*_*p*_^*2*^=.03.The interaction of Group and Valence was not significant, *F* (2,54)=.07, *p*=.933, *η*_*p*_^*2*^=.00. The interaction of Group and Electrode was not significant,*F*(2, 54)=1.40, *p*=.254, *η*_*p*_^*2*^=.05. The interaction of Valence and Electrode was also not significant,*F*(4, 108)=.81, *p*=.520, *η*_*p*_^*2*^=.03.

**Fig. 2:**
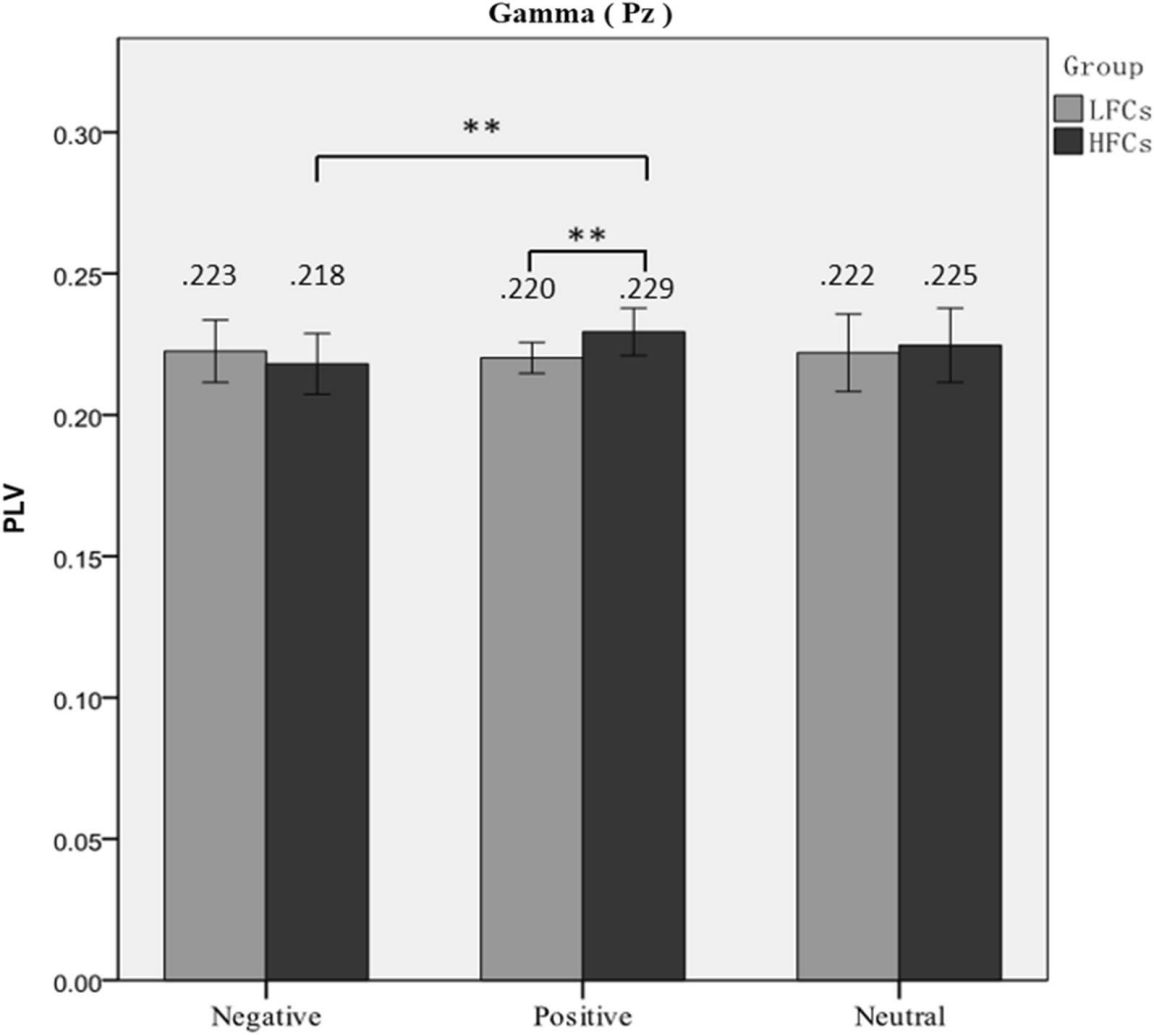
Gamma Interbrain Phase-locking-value (PLV) in the Parietal areas (Pz) between Low and High Family Cohesion Parent-adolescent Dyads (LFCs and HFCs) in Different Conditions. Note: *p < 0.05, **p < 0.01, ***p < .001

The interaction of Group ×Valence ×Electrodewas significant, *F*(4, 108)= 3.00, *p*=.022, *η*_*p*_^*2*^=.10.As shown in Figure 2, greater gamma interbrain synchrony was observed in the HFCs than the LFCs in the positive conditions at Pz(*t(*27)= 3.522, *p*=.002). At Pz, higher gamma interbrain synchrony was observed in the positive conditions than in the negative conditions in the HFCs. (*t(*27)= 3.319, *p*=.005).In the HFCs, higher gamma interbrain synchrony was observed at the Pz than at the Cz (*t(*13)= 2.368, *p*=.044) and Fz (*t(*13)= 2.039, *p*=.037) in positive conditions. There were no significant differences between different conditions at other electrode in the LFCs and HFCs (*ps*>.05).

To demonstrate the existence of neural synchronization between parents and adolescents when experiencing different emotion experience together, surrogate date was created similar to prior interbrain research [4]. Shuffling was performed by randomly forming dyads with parents and adolescents who were not from the same family. In this way, new 29-dyad samples were created. Mean PLV of the surrogate data was recalculated (*M* = .222 , *SD* = .005). In this case, we obtained an index of the gamma interbrain synchrony level that would be expected by chance. Using Wald-Wolfowitz test, we compared the real and surrogate data to assess the control distribution of the experimental effect. The PLV values in the Wald-Wolfowitz test were averaged across groups, valence conditions, and electrodes before the comparison. Results showed that there was significant difference in the distribution of the interbrain synchrony in the real parent-adolescent dyads and the random pairs (*p* = .017).

**Fig. 3:**
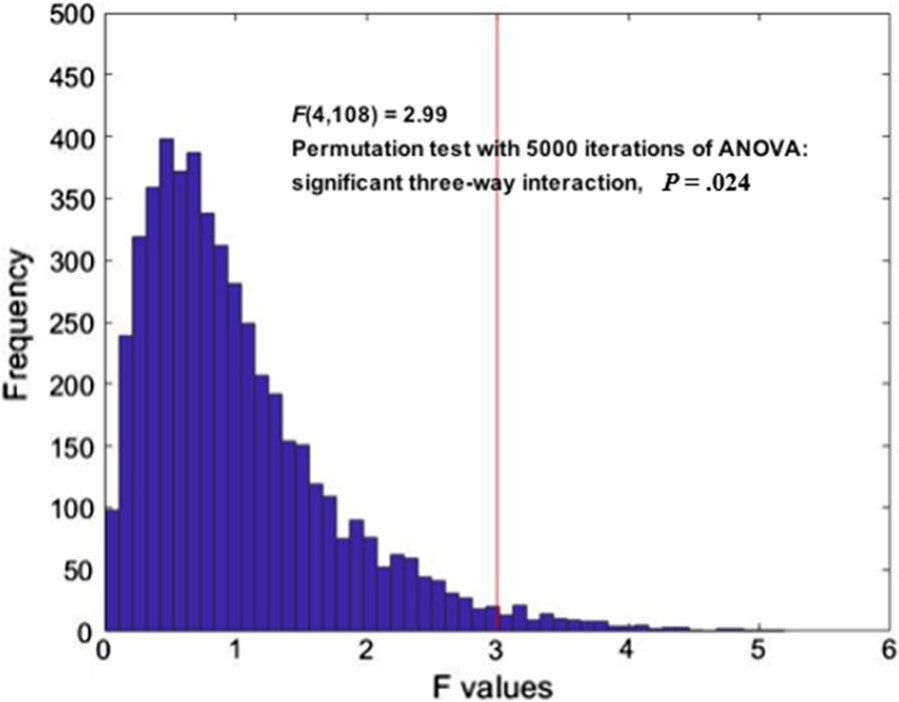
The results of the permutation test, showing the distribution of the interaction effect (F value). The interaction effect (red line) in real dyads was significant within the 5% area. The x-axis represents the F value, and the y-axis represents the number of the samples

Moreover, to validate the significant interaction effect in real dyads in the repeated measures ANOVA, a validation approach of the permutation test was applied. As mentioned, parent and adolescent dyads were randomly assigned to form new pairs who did not actually in a family. Repeated measures ANOVAs were performed for the surrogate data. Permutation test was conducted 5000 times to yield a distribution (*F* value), which was then compared with the original data. As shown in Figure 3, compared with the distribution generated by the permutation procedure, the interaction effect between Group × Valence × Electrode reached significance at the *p*< 0.05 level for the original pairs of the parent and adolescent dyads (*p*=.024).

**Fig. 4:**
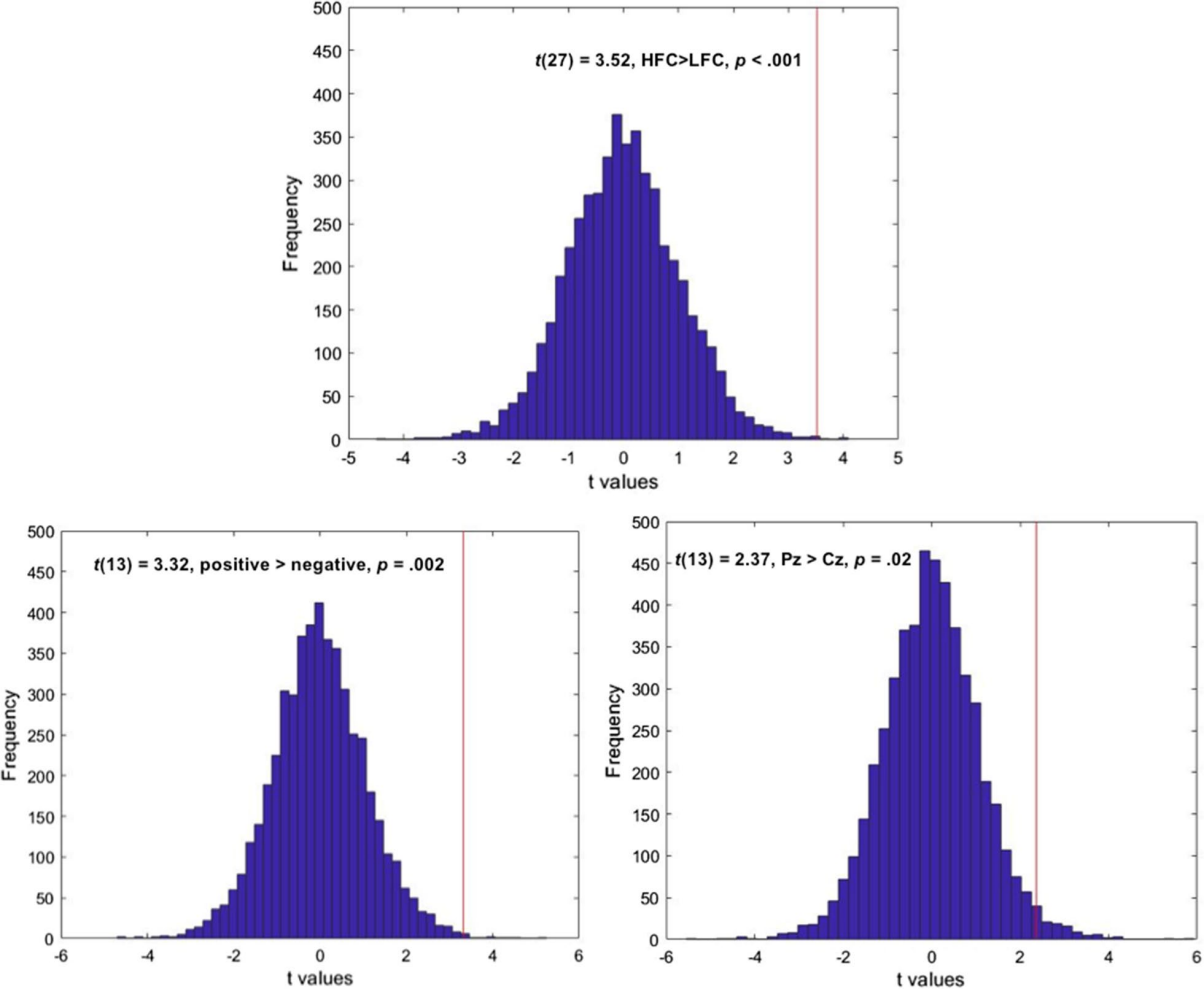
The results of the permutation test, showing the distribution of the simple effects (*t* values). The simple effects (red line) in real dyads were significant within the 5% area. The x-axis represents the *t* values, and the y-axis represents the number of the samples

Next, to validate the significant simple effects in real dyads in the above repeated measures ANOVA, a validation analysis was added by recruited pair randomization permutation test. The parent and adolescent dyads were shuffled as mentioned above. The permutation was conducted 5000 times to yield a null distribution of the PLV values for each group in different emotional conditions separately. Significant levels (*p*< 0.05) were assessed by comparing the PLVs from the original dyads with 5000 renditions of random pairs. As shown in Figure 4, results showed that the significant simple effects in real dyads in the repeated measures ANOVA survived after the random permutation test which was repeated 5000 times (group comparison at Pz under positive condition, HFC>LFC, *p* < .001; valence comparison at Pz for the HFC group, positive > negative, *p* = .002; electrode comparison under positive condition for the HFC group, Pz > Cz, *p* = .02). These results validated the existence of the differences in the neural synchronization between LFCs and HFCs, positive and negative conditions, and parietal and central regions when experiencing different emotion experience together.

## Discussion

Family cohesion has been regarded as an important component of family functioning. It reflects the affective and supportive relationship among family members [41]. Previous research has demonstrated the protective impact of family cohesion on the negative socio-emotional interactions (e.g., emotional conflicts) between parents and adolescents during “the period of storm and stress” [27]. The shared emotional stimulation between parent and child is proved to induce interbrain synchrony, implicating the inferring the mental states of others [2]. Nevertheless, there is less evidence has described how family cohesion is related to the synchronous activations and interbrain connectivity when experiencing emotions together between adolescent-parent dyads by using the neuroscientific method. The present study explored the differences in the interbrain synchrony between adolescent-parent dyads during synchronous positive and negative emotional stimulation with different family cohesion levels. By identifying the psychophysiological evidence for this relationship between family cohesion and the interbrain synchrony between parents and adolescents, we extended the understanding of the neural underpinnings of family cohesion that influence the social emotion perception and emotional sharing within family members.

Interbrain synchrony is demonstrated as a neural marker for social interaction. The moment-to-moment interactions between individuals’ brains can be understood as a bidirectional behavioral stimulus-to-brain coupling [28]. Even without the real information transmission and interaction, induced synchrony could be observed with the influence of common external stimuli according to the phase resetting theory (Burgess, 2001; [19]. In this case, the interbrain synchrony within the dyads in response to emotional film clips in our present study might reflect the degree of the emotional connection and the quality of shared attention between parents and adolescents during the socio-emotional processing at the neural level [45]. The simultaneous emotional stimulation might tap into the similar tendencies for emotional perception or responses across parents and adolescents. The results of the present study were consistent with the hypothesis that family cohesion is related to interbrain synchrony between the adolescent-parent dyads when experiencing emotions together. Specifically, we found that greater gamma interbrain synchrony in response to emotional film clips was observed in the HFCs than the LFCs in the positive conditions. However, there was no significant group difference between the HFCs and LFCs in the negative and neutral conditions. It is worth noting that the joint activity (viewing emotional film clips together) in the present study was passively with no face-to-face communication between dyads. The greater neural synchrony in the HFCs indicated that parents who have higher parental function were able to instinctively attune their mental state and align it with that of their child with minimal feedback in the form of behavioral cues [2]. Such interpersonal coordination is based on the strong emotional bonding and connection between the family members. The emotional bonding that family members experience toward each other is the core of family cohesion. Low level of family cohesion suggests emotional disengagement, less emotional closeness, and high independence. High level of family cohesion suggests emotional engagement, high emotional support and dependence (Olson, 1986). Family cohesion increases communication and emotional bonding with each other, which may promote their interbrain synchrony and coupling [3]. Moreover, previous research has found that children and adolescents in the positive emotional atmosphere families would tend to cooperate more efficiently and more emotional sharing with parents in positive emotional context compared with the individuals in negative atmosphere families [27]. This is maybe a possible explanation of the significant higher interbrain synchrony in HFCs compared with the LFCs in positive simulation conditions.

Also, we found a significant higher interbrain synchrony in HFCs in positive simulation conditions than in negative conditions. Previous research indicated emotional sharing and expressiveness is more apparent and easier for positive emotions than negative emotions, because of few social constraints regarding positive emotional expression [18]. In this case, when experiencing emotions together, positive emotional expression would be easier between children and parents, in which may result in a higher level of emotional sharing and interbrain synchrony within dyads. In a higher cohesive family, when the mother has a more emotional engagement, the child would be more willing to express positive emotions than negative emotions. The parents’ active participation will increase the child’s safety attachment, which may result in positive emotional sharing and expression to their parents [7, 14]. In this case, significant higher interbrain synchrony between high family cohesion adolescent-parent dyads could be observed in the positive emotional contexts rather than the negative emotional contexts.

In the current study, we only found high and low family cohesion adolescent-parent dyads differences in the gamma band interbrain synchrony indexed by the PLV in the parietal areas (Pz) when the dyads responded to emotional film clips. Parietal Gamma activities associated with emotion processing, including emotion recognition, emotion regulation and empathic sharing of others’ emotions. Gamma frequency bands in the frontal and parietal areas have been identified to provide discriminative information associated with emotion processing [33]. Parietal Gamma activity may be involved in emotional regulation, as this process integrates the top-down cognitive reappraisal process and the bottom-up sensory emotional process [25]. The right inferior parietal lobe is an important hub for both sensory-emotion integration and empathic sharing of others’ emotions [36]. In line with the previous study, findings from the present study have also demonstrated that functional communication between brains when experiencing emotions together (e.g., simultaneous emotional processing) relies on the oscillatory synchronization of gamma band responses [34, 37]. The gamma oscillations are functional and prominent in brain regions that underline emotional processing and functions (e.g., amygdala) [51]. For example, research indicated that increase in gamma band activity and its synchronization in the amygdala, prefrontal, and posterior cingulate cortices to emotional relative to neutral stimuli [35, 38]. When individual experiences negative social emotions (e.g., social threat), interbrain gamma synchrony associated with social coordination would be increased. Instead of triggering the alpha synchrony, the interbrain gamma synchrony may foster a shared emotional representation among individual and promote social coordination when facing negative emotional stimuli. [39]. Together with the results from previous studies, gamma interbrain synchrony as an important neural indicator of emotional sharing, the present study warrants further investigation of social emotion perception in this special age.

The present study examined the relationship between family cohesion and interbrain synchrony when experiencing emotions together, between adolescent-parent dyads. As the main limitation, the sample size of the present study is relatively small. It is necessary to include more adolescent-parent dyads in the study to increase the generalizability of our findings. To rule out the possible impact of relevant variables of the parent-adolescent emotional interactions on our findings, demographic variables, adolescents’ level of depression, adolescents’ level of anxiety, adolescents’ level of social support, parents’ level of depression, parents’ level of anxiety, and the level of parent involvement between the high and low family cohesion parent-adolescent dyads were examined. However, we didn’t include more variables about the parent-adolescent relationship (e.g., parenting style and family atmosphere), which might influence their social emotion perception and emotional sharing. It is important and necessary for future research to explore the impacts of other variables about the parent-adolescent relationship. Moreover, to increase the generalizability of our findings, longitudinal design as a potential approach is necessary to employ in future research to examine how the relationship between family cohesion and interbrain synchrony during emotional interactions accompanied by the brain development and maturity of adolescents. We found higher neural synchrony among parent-adolescent dyads with high family cohesion. However, the findings may only reflect an effect of interpersonal closeness on the neural synchrony. Since interpersonal closeness is an important criterion of family cohesion, we didn’t separate the measures of interpersonal closeness and family cohesion in the present study. In the future study, measuring interacting dyads with high interpersonal closeness and low family cohesion (e.g., peer dyads or teacher-student dyads) can be a possible way to rule out the possible effect of interpersonal closeness. Also, it is possible that the HFCs participants from different families may still respond more similarly to each other than to members of the LFCs group because of the naturally different brain response patterns. Therefore, neural synchrony between the HFC participants from different families or random groups (e.g., the dyads of HFC participant and stranger) need to be examined in future research.

Limitations notwithstanding, the findings of the current study contribute to the literature on deepening our understanding of the relationship between family cohesion and socio-emotional interaction for a special blood relationship in a special period -- adolescence. The current findings indicate that the impacts of family cohesion might be differently related to the gamma interbrain synchrony within dyads when sharing positive and negative emotions together. Since high interbrain synchrony of high family cohesion adolescents in the positive emotional situations reflected a higher quality of socio-emotional interactions, cultivating a positive family atmosphere and increase emotional sharing among family members may have important value in promoting social skills for adolescents. Last but not the least, our findings in gamma interbrain synchrony again demonstrated that examining gamma band activity and its synchronization could be a feasible way in the study of social emotion perception in different age groups (e.g., adolescents).

## Data Availability

The data are currently not publicly available due to participant privacy, but they are available from the corresponding author upon reasonable request.

## Appendix A

The averaged interbrain PLVs in the following four frequency bands were calculated for further statistical analysis: delta (1–3 Hz), theta (4–7 Hz), alpha (8–13 Hz), beta (14–30 Hz). Results of the 2*3*3 ANOVA on the interbrain phase synchrony in the delta, theta, alpha, beta bands were shown in Table.

See Tables 4 and 5

**Table 4:** Results of the ANOVA on the Delta, Theta, Alpha, Beta Interbrain Phase-locking-value *(PLV)*

PLV	Factor	*df*	*F*	*p*	*Partial η*^*2*^
Delta	Electrode	2,54	2.56	0.101	0.09
	Electrode*Group	2,54	0.02	0.964	0.00
	Valence	2,54	2.56	0.087	0.09
	Valence *Group	2,54	0.30	0.739	0.01
	Electrode*Valence	4,108	0.22	0.927	0.01
	Electrode*Valence*Group	4,108	2.60	0.040	0.09
	Group	1,27	0.01	0.929	0.00
Theta	Electrode	2,54	0.39	0.682	0.01
	Electrode*Group	2,54	0.93	0.402	0.03
	Valence	2,54	0.64	0.530	0.02
	Valence *Group	2,54	0.02	0.981	0.00
	Electrode*Valence	4,108	0.43	0.789	0.02
	Electrode*Valence*Group	4,108	1.89	0.118	0.07
	Group	1,27	0.096	0.760	0.00
Alpha	Electrode	2,54	4.51	0.015	0.14
	Electrode*Group	2,54	0.86	0.429	0.03
	Valence	2,54	1.93	0.156	0.07
	Valence *Group	2,54	0.88	0.422	0.03
	Electrode*Valence	4,108	0.38	0.823	0.01
	Electrode*Valence*Group	4,108	1.74	0.147	0.06
	Group	1,27	0.02	0.897	0.00
Beta	Electrode	2,54	2.59	0.085	0.09
	Electrode*Group	2,54	0.58	0.564	0.02
	Valence	2,54	0.51	0.606	0.02
	Valence *Group	2,54	0.88	0.42	0.03
	Electrode*Valence	4,108	1.06	0.380	0.04
	Electrode*Valence*Group	4,108	1.34	0.262	0.05
	Group	1,27	0.89	0.354	0.03

^4^. Of the interaction about group, only an Electrode*Valence*Group interaction in delta was significant, but post-hoc analysis did not find any significant difference. Therefore, the averaged interbrain PLVs in four frequency bands were not included in the main text. Delta, Theta, Alpha, Beta Interbrain Phase-locking-value (PLV) at Pz between Low and High Family Cohesion Parent-adolescent Dyads in Different Conditions were shown in Table

**Table 5:** Delta, Theta, Alpha, Beta Interbrain Phase-locking-value (PLV) at Pz between Low and High Family Cohesion Parent-adolescent Dyads in Different Conditio*ns*

PLV	Condition	LFCs (*M* ± *SD*)	HFCs (*M* ± *SD*)	*t(27)*	*d*	*p*	95%CI	
Delta	Positive	0.486 ± .022	0.489 ± 0.021	− 0.34	0.14	0.734	− 0.02	0.01
	Negative	0.482 ± 0.021	0.474 ± 0.017	1.19	0.42	0.244	− 0.01	0.02
	Neutral	0.481 ± 0.016	0.488 ± 0.027	− 0.94	− 0.32	0.355	− 0.02	0.01
Theta	Positive	0.482 ± 0.013	0.490 ± 0.011	− 1.68	− 0.07	0.105	− 0.02	0.00
	Negative	0.483 ± 0.013	0.488 ± .016	− 0.98	− 0.34	0.334	− 0.02	0.01
	Neutral	0.491 ± 0.020	0.489 ± 0.023	0.21	0.09	0.838	− 0.01	0.02
Alpha	Positive	0.381 ± 0.020	0.376 ± 0.022	0.55	0.24	0.587	− 0.01	0.03
	Negative	0.382 ± 0.022	0.379 ± 0.024	0.24	0.13	0.815	− 0.02	0.01
	Neutral	0.388 ± 0.016	0.385 ± 0.021	0.42	0.16	0.675	− 0.03	0.00
Beta	Positive	0.219 ± 0.007	0.213 ± 0.010	1.52	0.70	0.141	-− 0.01	0.00
	Negative	0.218 ± 0.011	0.217 ± 0.011	0.18	0.09	0.855	− 0.00	0.01
	Neutral	0.216 ± 0.007	0.212 ± 0.011	1.10	0.43	0.279	− 0.01	0.01

*LFCs* low family cohesion parent-adolescent dyads, *HFCs* high family cohesion parent-adolescent dyads

5.

## Appendix B

To rule out the possible impact of relevant variables of the parent-adolescent emotional interactions on our findings, demographic variables, adolescents’ level of depression, adolescents’ level of anxiety, adolescents’ level of social support, parents’ level of depression, parents’ level of anxiety, and the level of parent involvement between the high and low family cohesion parent-adolescents dyad were examined. Repeated measures ANOVAs were used and these relevant variables were set as the covariates. The results of the repeated measures ANOVA indicated that there were no significant main effects of most of the demographic variables and other examined variables on our Gamma Interbrain PLV between the high and low family cohesion group. Therefore, demographic variables and other examined variables were not included in subsequent analyses.

Adolescent’s age: F(1, 26) = 3.57, p = 0.070, ηp2 = 0.12; parent’s age: F(1, 26) = 0.07, p = 0.798, ηp2 = 0.00; adolescent’s gender: F(1, 26) = 3.53, p = 0.071, ηp2 = 0.12; parent’s gender: F(1, 26) = 1.03, p = 0.319, *η*_*p*_^*2*^ = 0.04; parent-adolescent relations vary by gender (i.e., mother-daughter, mother-son. father-daughter, father-son): *F*(1,26) = 3.83, *p* = 0.061, *η*_*p*_^*2*^ = 0.13; only child: *F*(1, 26) = 0.15, *p* = 0.705, *η*_*p*_^*2*^ = 0.01; father’s education level: *F*(1, 26) = 2.20, *p* = 0.150, *η*_*p*_^*2*^ = 0.08; mother’s education level: *F*(1, 26) = 0.79, *p* = 0.383, *η*_*p*_^*2*^ = 0.03; adolescent’s depression: *F*(1, 26) = 0.43, *p* = 0.517, *η*_*p*_^*2*^ = 0.02; adolescent’s anxiety: *F*(1, 26) = 3.12, *p* = 0.089, *η*_*p*_^*2*^ = 0.11; parent’s depression: *F*(1, 26) = 0.04, *p* = 0.841, *η*_*p*_^*2*^ = 0.00; parent’s anxiety: *F*(1, 26) = 0.22, *p* = 0.64, *η*_*p*_^*2*^ = 0.01; parent involvement: *F*(1, 26) = 0.14, *p* = 0.709, *η*_*p*_^*2*^ = 0.01.

## Abbreviations

EEG: Electroencephalograph
LFCs: Low family cohesion parent-adolescent dyads
HFCs: High family cohesion parent-adolescent dyads
PLV: Phase-locking-value
FACESII-CV: The Family Adaptability and Cohesion Scale, Chinese Version
